# Adoption of a Societal Perspective in Economic Evaluations of Musculoskeletal Disorders: A Conceptual Paper

**DOI:** 10.3390/jmahp12030018

**Published:** 2024-08-12

**Authors:** Francis Fatoye, Tadesse Gebrye, Leo Nherera, Paul Trueman

**Affiliations:** 1 Department of Health Professions, Faculty of Health and Education, Brooks Building I Manchester Metropolitan University, Manchester M15 6GX, UK; t.gebrye@mmu.ac.uk; 2 Smith + Nephew Inc., Global Market Access, 5600 Clearfork Main St, Fort Worth, TX 76109, USA; leo.nherera@smith-nephew.com (L.N.); paul.trueman@smith-nephew.com (P.T.)

**Keywords:** perspective, societal perspective, health economic, economic evaluation

## Abstract

Economic evaluations are used to compare the costs and consequences of healthcare interventions, including those for musculoskeletal (MSK) disorders, which are very common and a major source of morbidity and absence from work. Reimbursement decisions for interventions for MSK disorders by decision-makers rely on the findings of economic evaluations, the design and results of which depend largely on the perspective adopted. Despite methodological advancements in economic evaluations, there are no clear guidelines on the perspective to adopt. This paper explores the adoption of a societal perspective in economic evaluations of MSK disorders. Within health economics evaluations, the most commonly used perspectives include the payer perspective, the healthcare perspective, and the societal perspective. To facilitate optimal resource allocation decisions in order to reduce the significant economic burden of MSK disorders and improve the health outcomes of individuals with these disorders, all costs and benefits associated with interventions for them should be included. Thus, the societal perspective is arguably a preferable option to the others for economic evaluations of interventions for MSK disorders.

## 1. Introduction

Musculoskeletal (MSK) disorders include more than 150 diagnoses that affect the locomotor system; these conditions are characterised by pain and reduced physical function [1]. Each year, 20% of people in the UK see a doctor about an MSK problem [2]. MSK disorders are among the most common causes of long-term incapacity for work, sickness absences, and early retirement [3]. Worldwide, the total number of MSK disability-adjusted life years (DALYs) increased significantly from 80,225,635 in 2000 to 107,885,833 in 2015 [4]. People with MSK disorders are less likely to be in work than those with long-term health conditions [5], with data indicating that around 38% of working-age adults with an MSK condition in the United Kingdom (UK) are out of work, compared to 19% of people with long-term health conditions [6]. A recent report by the European Agency for Safety and Health at Work (EU-OSHA) also showed that more than half of European workers experience MSK disorders [7].

There are significant economic consequences associated with MSK disorders and the associated work-related disability, including workers’ compensation payments, absenteeism from the workforce, and presenteeism resulting in reduced participation at work [8]. In 2012, MSK disorders in the United States cost approximately USD 213 billion in direct and indirect costs, or 1.4% of the United States’ gross domestic product (GDP) [9]. MSK disorders accounted for the third-largest area of English National Health Service (NHS) program spending, at GBP 5 billion, in 2013–2014 [10]. Direct costs commonly include costs incurred for physician services, medical devices, medications, hospital services, diagnostic tests, and caregivers. Indirect costs include all types of productivity loss, i.e., temporary or long-term inability to work [11]. Estimates of the direct and indirect costs of MSK disorders in different countries vary greatly. However, in most studies that used human capital approaches, indirect costs represented a greater proportion of the total burden of illness than the costs incurred by the health service [12]. A recent review paper on the economic burden of rheumatoid arthritis found that indirect costs accounted for between 39% and 86% of the total cost, reflecting the chronic, long-term nature of the condition [13]. Indirect costs remain significant in the case of acute MSK injuries, such as rotator cuff tears in the shoulder, where a recent study suggested that lost productivity makes up between 15 and 20% of the total cost in the 12 months following the injury [14]. Furthermore, at a global scale, the economic burden associated with MSK disorders was attributed to high body mass index in 2019, where the burden accounted for 0.2% of the global gross domestic product [15]. According to Chen et al., the global total costs of musculoskeletal disorders reached USD 180.7 billion, more than 66% of which was productivity losses.

Given the significant costs associated with MSK conditions, interventions to help reduce, delay, or repair such conditions are often found to be cost-effective [16]. Preventative interventions to reduce or delay the risk of developing MSK conditions are highly cost-effective [16]. A report commissioned by Public Health England examined the return on investment of seven interventions designed to prevent and/or treat MSK conditions, including risk assessment for back pain, self-referral to a physiotherapist, and rehabilitation for knee pain. The study found a beneficial return on investment for four of the seven interventions, with self-referral to a physiotherapist offering potential savings of GBP 98 for each GBP 1 spent on healthcare provision [16]. When indirect costs were included in the analysis, all seven interventions were reported to offer a positive return on investment.

Similarly, the evidence on surgical interventions for knee and hip osteoarthritis also suggests that these are cost-effective [17]. The study identified that cost-effectiveness varied depending on factors such as disease severity, the timing of the intervention, and patient age [17]. Furthermore, significant variations were observed in the assumptions made to estimate the indirect costs of some MSK conditions, such as lower back pain, attributable to lost productivity [17]. However, on the whole, surgical intervention was found to be cost-effective compared to non-surgical alternatives [17]. 

The reviews of economic evidence repeatedly highlight the importance of perspective in economic evaluations. The perspective helps to identify which costs and benefits are considered in an economic evaluation, and it not only plays a critical role in the design of the study but can also have a significant influence on determining whether an intervention should be considered cost-effective and recommended for widespread use. The purpose of this conceptual paper is to explore the adoption of a societal perspective in economic evaluations of MSK disorders [17]. 

## 2. Perspectives in Economic Evaluations and Health Technology Assessment Submissions

Economic evaluations help to guide the allocation of scarce healthcare resources. This helps us to compare the efficiency of alternative interventions, and the perspective of economic evaluations is a key dimension [18] that should be determined before the evaluation begins [19]. The design, analysis, and reported results of economic evaluations usually depend on the chosen perspective [20]. The most commonly used perspectives in economic evaluation include the payer perspective, the healthcare perspective, and the societal perspective [20]. The perspective adopted, in turn, determines the types of costs and effects included in the economic evaluations. The healthcare perspective considers costs that are accrued within the healthcare sector, such as the costs of diagnosis and treatment. A payer perspective is a narrower version of the healthcare perspective that considers only those costs that fall on a payer; for example, in some healthcare systems, this would exclude any co-payment contribution made by the patient. The societal perspective considers all of the costs and effects that are accrued, regardless of who experiences them. This would include costs and benefits that may fall outside the healthcare system, including those incurred by the patient and society more broadly, such as the impact on productivity. In the societal perspective transfer are not considered as they are neutralised at societal perspective. We do acknowledge that estimating the indirect costs has its challenges. For instance, incomplete data and the quality of the data can determine how accurate the estimates are. A heterogeneous population also means that different groups can place different estimates of health benefit on the assessed interventions, making it difficult to accurately estimate indirect costs. 

Best practice principles for economic evaluations have typically recommended the use of a societal perspective to provide a holistic determination of the value of an intervention. One of the most definitive best practice guidelines, the recommendations of the Second Panel on Cost-Effectiveness in Health and Medicine, explicitly called for both societal and payer perspective that identifies relevant costs and outcomes associated with an intervention, stating ‘*The major categories of resource use that should be included are costs of health care services; costs of patient time expended for the intervention; costs associated with caregiving (paid or unpaid); other costs associated with illness, such as child care and travel expenses; economic costs borne by employers, other employees*.’ [21]. Nevertheless, it is important to note that the Australian Commonwealth Department of Health, Housing, and Community Services [22] and the Ontario Guidelines for Economic Analysis of Pharmaceutical Products [23] suggest that the healthcare sector and/or societal perspective be adopted in health economic analysis. 

Several consensus papers have also suggested adopting a societal perspective in the evaluation of healthcare interventions in MSK conditions, highlighting the importance of capturing the substantial indirect costs associated with these conditions [24,25]. The OMERACT Working Group produced guidelines for economic evaluation in rheumatoid arthritis, including reference to capturing ‘all direct medical and non-medical costs’ in the analysis but reporting indirect costs (i.e., productivity losses) separately [24]. Similarly, consensus-based guidelines relating to economic evaluations of osteoporosis, osteoarthritis, and musculoskeletal diseases recommend the use of both societal and healthcare perspectives [25]. 

Many health technology assessment (HTA) agencies use cost-effectiveness analysis to make recommendations regarding the acceptance, restriction, or rejection of interventions, where cost-effectiveness is expressed using the incremental cost-effectiveness ratio (ICER) [26]. However, most HTA guidelines are inadequate in providing clear guidance on what to include under different perspectives [18]. 

HTA agencies such as the State Institute for Drug Control (SUKL) of the Czech Republic, the Italian Medicines Agency, and the Pharmaceutical Management Agency (Pharmac) of New Zealand adopt a narrow budget perspective that excludes consideration of costs and consequences accrued outside the health system, such as those falling to patients, carers, and employers [18]. However, the guidelines from HTA agencies based in countries with multi-payer systems are more likely to consider a societal perspective explicitly. For example, Australia [27], Canada [28], the Netherlands [29], Germany [30], and Sweden [31] suggest that the costs incurred by the government, caregivers, patients, private healthcare providers, and the public healthcare system should be included under the societal perspective.

However, economic evaluations of MSK disorders are inconsistent in the perspective adopted. A systematic review that assessed existing evidence on the cost-effectiveness of surgical interventions for the management of knee and hip osteoarthritis indicated that, out of the 23 studies included in the review, 8 (35%), 8 (35%), and 3 (13%) were conducted from the healthcare system, societal, and healthcare and societal perspectives, respectively [32]; however, the perspective used in the remaining 4 (17%) studies was not mentioned. The key cost drivers within MSK economic analyses were reported to be the grade of treating clinicians and the specific consultation length for clinical visits, which are important to determine true patient-level costs [32]. 

The choice of perspective is more than an academic consideration, given that determining whether an intervention is cost-effective can influence the degree to which it is made available to patients and reimbursed. The next section considers an applied example of how adopting a healthcare perspective or a societal perspective can fundamentally change the conclusion of a health economics evaluation. 

## 3. Illustrative Case Studies: The Impact of Adopting Alternative Perspectives in Economic Evaluation

Economic evaluations of the surgical repair of rotator cuff tears illustrate how the choice of a healthcare or societal perspective can have a fundamental impact on cost-effectiveness ratios and, ultimately, on whether an intervention should be considered for widespread adoption [33]. Rotator cuff tears (RCTs) are among the most common causes of shoulder pain and are a leading cause of productivity loss [14]. There has been a substantial increase in the volume of operative interventions for rotator cuff tears using arthroscopic surgical closure [33], with or without additional advanced technologies, such as augmentation with a bioinductive collagen implant [34,35] or a subacromial spacer [36]. The ability to heal a cuff tear after surgery is impacted by age, obesity, and other risk factors [37]. 

The cost-effectiveness of surgical repairs of RCTs was first considered by Vitale et al. in 2007 [38]. A cost–utility analysis was conducted in the United States of America based on a prospective study of surgical closure. A societal perspective was adopted, including costs of all services associated with providing care to patients, regardless of who bears the costs. As cost data were derived from hospital charges, some attempts were made to reconcile costs and charges, as the latter are typically higher and are not necessarily a fair reflection of the actual resources utilised in the provision of care. No attempt was made to capture indirect costs, with the authors suggesting that these would be reflected in the quality adjusted life years (QALY) values. Utilities were derived using both the Health Utilities Index (HUI) and the EuroQol EQ-5D, generating two alternative cost–utility estimates. The study was designed to generate an estimate of the cost and utility of rotator cuff surgery, as well as to explore the impact of adopting alternative approaches to capture utility scores. 

The outcomes of the study [38] showed a favourable cost-effectiveness ratio for rotator cuff surgery, although there were significant variations in the outcomes depending on the choice of utility measure. Using the HUI data, the incremental cost per QALY was USD ≈ 13,000/QALY whilst using the EQ-5D data generated a lower estimate of USD ≈ 3000/QALY. In both cases, the authors concluded that rotator cuff surgery can be considered cost-effective, falling below the threshold of USD 50,000/QALY that is widely accepted in the United States. 

A subsequent study by Mather et al., in 2013 [39], examined the value of surgical treatment for full-thickness RCTs from a societal perspective. Similar to Vitale’s study, Mather et al. considered the value of surgical repair for rotator cuff tears compared to non-operative treatment using a cost–utility analysis. The utility values in Mather et al. study was derived from the Short Form-12 (SF-12). The costs of surgical repair were similar in the two studies; Vitale et al. reported a cost of USD 18,924 (USD 10,605 when adjusted from charges to costs), compared to USD 15,063 in Mather’s analysis. Utility benefits associated with successful surgery varied between the studies. Vitale et al. reported an incremental QALY gain of between 0.81 and 3.43, depending on the choice of utility instrument, whilst Mather et al. reported a benefit of 0.62 QALYs in favour of surgical intervention. The main difference between the studies was the perspective adopted and the handling of indirect costs, which were excluded from Vitale et al.’s study but included in Mather et al.’s study. The latter sought to estimate indirect costs, including missed workdays and disability payments, weighting these by household income and the probability of being in employment. The results of Mather et al.’s study show that rotator cuff surgery was considered dominant and that it generated improved outcomes (incremental QALYs +0.62) at a lower total cost. 

Subgroup analyses conducted by Mather et al. further illustrated the impact of indirect costs. Surgical intervention was cost-saving compared to non-operative interventions in patients aged up to 61 years. In older age groups, surgery was cost-additive, due to lower rates of employment and lower expected productivity costs. Despite this, surgery remained cost-effective across all age groups. 

**Figure 1:**
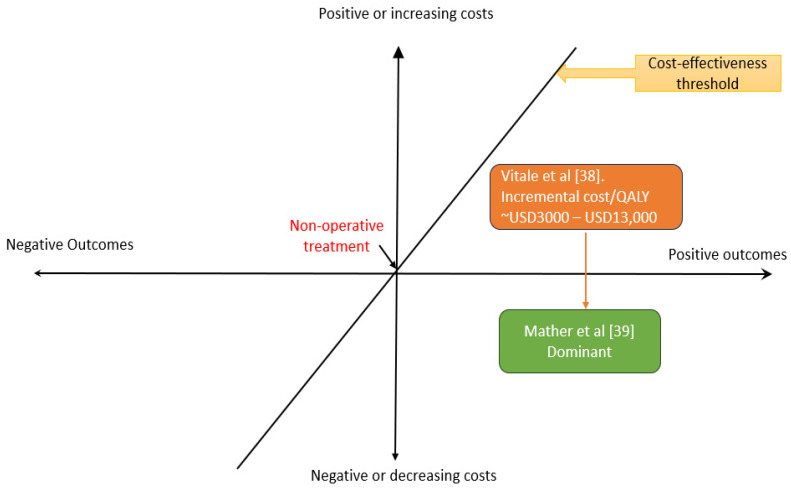
A decision plane showing cost-effective results payor perspective [38] and dominant results societal perspective [39].

These studies neatly illustrate the impact of considering the societal perspective in economic evaluations of MSK conditions. By including the societal costs, the cost-effectiveness of rotator cuff surgery shifted from the top-right quadrant of the cost-effectiveness plane (better outcomes at higher cost) to the bottom-right quadrant (dominant) (Figure 1). 

This is particularly important from a healthcare decision-making perspective. Healthcare interventions and procedures in Australia follow a standardised procedure to decide which services in medical care are reimbursed [27]. The Medical Services Advisory Committee (MSAC), an independent non-statutory committee established by the Australian Government Minister, advises the Minister for Health on the listing of the Medicare Benefits Schedule subsidies for orthopaedic technologies other than prostheses. The key terms considered by the committee when advising the Minister of Health are ‘the strength of evidence in relation to the comparative safety, effectiveness, cost-effectiveness, and total cost of the medical service’ [40]. This principle is adopted by many payers who seek to determine whether innovative technologies can provide improved outcomes at lower costs before agreeing to cover their costs. Similarly, in its Medical Technologies Evaluation Programme, NICE only considers technologies with the potential to offer better outcomes at lower costs for medical technology guidance [41]. The NICE methods manual highlights that medical devices that are cost-saving/cost-neutral will be routed through the medical technologies guidance route, whilst those that are cost-additive are likely to be subject to full technology appraisal [42]. At the provider level, introducing cost-saving/neutral technologies is far easier to accommodate in a constrained budget, whereas cost-additive technologies mean that other service lines need to be reduced/stopped in order to release the necessary funds.

In both of these cases, the evidence offered by Vitale et al. [38], based on a healthcare perspective, may have resulted in restrictions on the use of rotator cuff surgery if the ICER as in some cases had fallen above the threshold, whilst the evidence provided by Mather et al. would have supported more widespread adoption. This illustrates how the choice of perspective can have a real impact on patients’ access to new technologies.

## 4. Conclusions

From the evidence presented above, it is clear that the healthcare perspective is commonly used in economic evaluations of interventions for MSK disorders. A recent systematic review on the cost-effectiveness of surgical interventions for the management of osteoarthritis revealed that, out of the 23 included studies, many [9] of them adopted a healthcare perspective [17]. However, adopting a societal perspective for economic evaluation could lead to potential cost savings from the evaluated interventions [43]. The costs of lost productivity in individuals with MSK disorders can exceed healthcare costs; as such, economic evaluations conducted from a healthcare perspective provide a partial analysis of the value of these interventions [44]. Therefore, any economic evaluation that does not consider productivity loss and/or return to work will not fully capture the costs and benefits associated with such interventions. Health economics is founded on welfare economics, which indicates that an economic evaluation should include the impact of an intervention on the whole of society [45]. Hence, it is important to have clear guidance on how to value lost productivity in economic evaluations of interventions for MSK disorders. Twenty-two of the thirty national pharmacoeconomic guidelines identified recommend performing economic evaluations using the societal perspective [46]. To facilitate optimal resource allocation decisions, reduce the significant burden of MSK disorders, and improve the health outcomes of individuals with these disorders, all costs and benefits of interventions for them should be included, regardless of who incurs the costs [47]. Therefore, it is hereby recommended that the societal perspective, which captures all associated costs in health economics evaluations of interventions for MSK disorders, should be adopted as the primary perspective, with a healthcare perspective reported as a secondary outcome. Otherwise, the findings of such evaluations may underestimate the costs associated with specific interventions for these disorders and may result in suboptimal resource allocation, thereby incurring losses in the total welfare of society.
